# Association of trans fatty acids with lipids and other cardiovascular risk factors in an Indian industrial population

**DOI:** 10.1186/s13104-019-4352-7

**Published:** 2019-06-17

**Authors:** Ruby Gupta, Ransi Ann Abraham, Dimple Kondal, Savita Dhatwalia, Panniyammakal Jeemon, K. S. Reddy, D. Prabhakaran, Lakshmy Ramakrishnan

**Affiliations:** 1 Public Health Foundation of India, Gurgaon, India; 2 Department of Cardiac Biochemistry, Cardio-thoracic Centre, All India Institute of Medical Sciences, New Delhi, India; 3 Sree Chitra Tirunal Institute for Medical Sciences and Technology, Trivandrum, Kerala India

**Keywords:** Saturated fatty acid, Monounsaturated fatty acid, Polyunsaturated fatty acid, Trans fatty acid, hs CRP, Lipids

## Abstract

**Objective:**

Trans-fat, an invariable component of industrial fat is considered as one of the major dietary factors associated with CVD. Although the use of trans-fat is completely banned in some of the high-income countries where the CVD epidemic is declining, it is widely used in LMIC. We aimed to investigate the association of trans fatty acid in serum with risk markers of CVD in an industrial population in India. Participants were randomly selected from a study conducted in an industrial setting among employees and their family members. Information related to their demographic profile, anthropometric measurements, oil intake were recorded. Fasting samples were collected and stored at − 80 °C for analysis. Their lipid profile and hs CRP were measured and fatty acids analyzed using gas chromatography (GC) with flame ionization detector (FID).

**Results:**

Complete data was available for 176 participants. Among trans fatty acids, mono trans fatty acid was significant predictor of serum triglycerides [Unadjusted β (95% CI) 22.9 (2.6, 43.2); Adjusted β (95% CI) 20.4 (3.5, 37.3)]. None of the other trans fatty acids either individually or in group correlated with any of the biochemical markers studied.

**Electronic supplementary material:**

The online version of this article (10.1186/s13104-019-4352-7) contains supplementary material, which is available to authorized users.

## Introduction

Cardiovascular Disease (CVD) burden is increasing in low and middle-income countries [1] in the past two decades. Studies imply that consumption of trans fatty acids is directly contributing to the twentieth century epidemic of cardiovascular disease [2]. Several prospective epidemiological studies provide strong evidence on the association of trans-fat consumption and coronary heart disease [3–5]. Adverse effects of trans fatty acids are mainly due to their effects on lipid levels, endothelial function and inflammation [6]. The increase in cardiovascular disease risk in developing countries like India is largely due to sociological changes, urbanization and socio-economic status that influences pattern of dietary fat intake. India is a country with wide variation in food habits and trans fatty acids from dietary sources may be contributing to increased risk of CVD. In many parts of India, hydrogenated vegetable oil in the form of Vanaspati are consumed in great quantity which is known to have highest content of trans fat. On the other hand, in northern India the most commonly used oil is mustard oil that is rich in alpha-linoleic acid (18:3, n − 3), a polyunsaturated fatty acid. In a recent study, high trans-fat content was reported in fried food and snacks [7, 8] as well as edible oils from various commercially available brands in India [9].

Intake of fats can be best assessed by objectively measuring them in serum/plasma under controlled conditions [10]. Recently, WHO has announced an initiative called REPLACE to eliminate trans fat completely from the world by 2023. To assess the intake of trans fat and the gravity of problem caused by trans fat in India we need to objectively measure trans fat in blood [11]. To the best of our knowledge, information on levels of trans fatty acids in plasma/serum are not available from resident Indians. In the present study we estimated serum fatty acid levels in an industrial population from India and evaluated its correlation with other risk factors for CVD, like lipids, lipoproteins and markers of inflammation.

## Main text

### Methods

The present study was part of a larger study being undertaken in an industrial population from Delhi [12]. The study was conducted to assess the impact of a risk reduction programme on risk factor clustering in selected industrial population. We randomly selected 200 individuals from the baseline survey of the main study, conducted in an Industry in periphery of New Delhi. Participants were industry employees and their family members, between the age 20 to 69 years. Individuals who reported smoking (more than 10 cig/day), having heart disease, stroke, chronic renal failure, thyroid disorder and cancer were excluded from the study. Participants with diabetes (history or freshly diagnosed) were not included in the study. Also, those who reported consuming any dietary supplements or medications known to alter lipids, on steroids and beta-blockers were excluded.

Data related to their demographic profile, anthropometric measurements, frequency of food intake and type of oil use were collected for all the individuals participating in the study. Blood samples were collected after an overnight fast of 10–12 h. Serum was separated and stored at − 70 °C for analysis of lipids and fatty acids. Total cholesterol was estimated by CHOD-PAP (cholesterol oxidase/*p*-aminophenazone) method, triglycerides by GPO-PAP (glycerol phosphate oxidase-peroxidase aminophenazone) method, HDL-c was estimated by the precipitation method using phosphotungstate/magnesium precipitation of apolipoprotein B containing lipoproteins followed by estimation of cholesterol in the supernatant by enzymatic method, using kits from RANDOX. Apo A1, Apo B were estimated by the immunoturbidimetry method and hs CRP was estimated by latex enhanced agglutination turbidimetry using kits from Sentinel Diagnostics (Milan, Italy).

#### Fatty acid analysis

Esterification of fatty acids in serum was performed using method of [13]. 200 µL serum was mixed with 2 mL of methanol-toluene (4:1, v/v) containing 0.02 g 21:0/L and 0.12 g butylated hydroxytoluene/L followed by drop wise addition of 200 µL acetyl chloride and heating to 100 °C for 1 h. The reaction was stopped by adding 5.0 mL of 6% potassium carbonate. The sample was centrifuged at 3000 rpm at 4 °C for 10 min, the toluene layer was separated and evaporated under stream of nitrogen. Fatty acid methyl esters were re-dissolved in iso-octane and were subjected to gas–liquid chromatography. Column used was fused silica capillary SP2560, 100 m × 250 µm internal diameters × 0.20 µm film (Supelco, Bellefonte, Pennsylvania). The port temperatures of both the injector and the detector were set at 250 °C. The Oven temperature was initially set at 170 °C for 2 min and then increased 10 °C/min until a temperature of 190 °C is reached and then held for 1 min. Temperature was then increased at the rate 3 °C/min until 220 °C is reached and maintained. 1 µL sample was used for injection with a split of 10:1. The chromatograph was equipped with a flame ionization detector. Fatty acid peaks were identified by comparing their retention times with those of a standard mixture from Supelco (SIGMA-ALDRICH) using software WINACDS 7.0 from AIMIL, Nucon Technologies.

#### Statistical analysis

The distribution of the parameters was assessed graphically using histograms, q–q plots and Shapiro-Wilks lambda test. Data are presented as mean ± SD for normally distributed and as median (Inter-quartile range) for non-normally distributed fatty acids. Individual fatty acids were expressed in terms of percentage of total fatty acids measured. The strength of relationship between lipid profile and various fatty acids were calculated using Pearson/Spearman rank correlation whichever appropriate. Linear regression was done to assess the strength of association of total cholesterol, triglycerides, HDL-c, LDL-c, TC/HDL ratio, Apo A1, Apo B and hs CRP (as outcomes) with various fatty acids (Independent variables). The model was also adjusted for age, gender, BMI, diet, smoking and alcohol use. The β estimates with 95% confidence intervals are reported. All the statistical analysis was done using STATA 15.1 version (College station, Texas, USA).

Trans fatty acids were further grouped as mono trans fatty acids: C16:1 *t*, C 18:1 *9t*, C 18:1 *11t*; poly trans fatty acids: C 18:2 *t*, C 18:3 *t*; total trans fatty acid: mono trans + poly trans.

### Results

Total 1229 employees were recruited for the main study. After screening for the exclusion criteria, 518 individuals were found eligible. We randomly selected 200 individuals for our study and complete biochemical as well as fatty acid profile were available for 176 individuals. The mean age of the study population was 47.23 ± 6.0 years and were predominantly males (89.2%). The mean Body Mass Index (BMI in kg/m^2^) of the participants was 24.88 ± 3.43 with a mean waist circumference of 91.50 ± 9.56 cm. 40% of the participants were vegetarian, 22% were smokers and 35% were current users of alcohol. 9% of the study participant had self-reported hypertension. The basic characteristics of the study participants, their biochemical risk markers are provided in Table 1. The mean percent content of trans fatty acid in the study participants were highly skewed as can be seen in Additional file 1: Table S1.

**Table 1:** Basic characteristics and biochemical markers of the study participants (n = 176)

Parameters	N (%)/mean (SD)/median (IQR)
Age in years, mean (sd)	47.2 (6.0)
Gender, males (%)	157 (89.2)
BMI (kg/m^2^), mean (sd)	24.9 (3.4)
Waist circumference (cm), mean (sd)	91.5 (9.5)
Vegetarian diet, n (%)	71 (40.3)
Ever smokers, n (%)	38 (21.6)
Alcohol use, n (%)	62 (35.2)
Hypertension, n (%)	16 (9.1)
Cholesterol (mg/dL), mean (sd)	194.7 (30.7)
Triglycerides (mg/dL), mean (sd)	139.5 (62.4)
HDL-c (mg/dL), mean (sd)	37.5 (9.3)
Chol/hdl ratio, mean (sd)	5.4 (1.0)
LDL-c (mg/dL), mean (sd)	130.8 (28.7)
Apo A_1_ (mg/dL), mean (sd)	125.8 (23.7)
Apo B (mg/dL), mean (sd)	106.9 (20.0)
Hs CRP (mg/L), median (IQR)	1.7 (0.8, 3.8)

BMI, body mass index; IQR, inter quartile range; HDL-c, high-density lipoprotein cholesterol; LDL-c, low-density lipoprotein cholesterol; Apo A1, apolipoprotein A1; Apo B, apolipoprotein B; hs CRP, high sensitive c-reactive protein

Table 2 shows the correlation between trans fatty acids with other biochemical risk markers of CVD. Among trans fatty acids, mono trans fatty acid was positively associated with serum triglycerides (r = 0.166) and C16:1 trans with Apo B (r = 0.153) and LDL (r = 0.112). C18:2 trans was inversely associated with HDL levels in serum (r = − 0.136). The relationship between trans fatty acids and lipids and other markers remain the same after adjustment with age and gender (data not shown). None of the other trans fatty acids either individually or in group correlated with any of the biochemical markers studied.

**Table 2:** Correlation coefficient between trans fatty acids with lipids and inflammatory marker

Fatty acids	TC	TG	HDL	TC/HDL ratio	LDL	Apo A1	Apo B	hs CRP
C18:1 *trans*	− 0.086	0.091	− 0.071	0.003	− 0.143	0.041	− 0.117	− 0.037
C18:2 *trans*	− 0.077	− 0.041	− 0.136	0.095	− 0.041	− 0.111	− 0.066	− 0.073
C18:3 *trans*	− 0.003	0.011	0.088	− 0.112	− 0.008	− 0.078	− 0.036	0.055
Mono *trans*	− 0.014	0.166	− 0.105	0.063	− 0.018	0.013	0.028	− 0.056
Poly *trans*	− 0.037	− 0.009	0.014	− 0.052	− 0.024	− 0.114	− 0.060	0.016
Total *trans*	− 0.038	0.059	− 0.029	− 0.020	− 0.029	− 0.098	− 0.043	− 0.008
C16:1 *trans*	0.056	0.143	− 0.078	0.082	0.112	− 0.021	0.153	− 0.041
C 18:1 *11 trans*	− 0.099	0.073	− 0.006	− 0.070	− 0.104	− 0.077	− 0.054	− 0.028
C18:1 *9 trans*	− 0.045	0.065	− 0.078	0.040	− 0.107	0.090	− 0.106	− 0.028

TC, total cholesterol; TG, triglycerides; HDL, high density lipoprotein cholesterol; LDL, low-density lipoprotein cholesterol; 18:1 trans, C 18:1 9t + C 18:1 11t; mono trans fatty acids, C16:1 t + C 18:1 9t (Elaidic acid) + C 18:1 11t (Vaccenic acid); poly trans fatty acids, C 18:2 t + C 18:3 t; total trans fatty acid, mono trans + poly trans; Apo A1, apolipoprotein A1; Apo B, apolipoprotein B; hs CRP, high sensitive c-reactive protein

On regression analysis (Tables 3, 4, 5), mono trans and trans palmitoleic acid significantly predicted high triglyceride levels even after adjustment with potential confounders [Unadjusted β (95% CI) 22.9 (2.6, 43.2); Adjusted β (95% CI) 20.4 (3.5, 37.3) and Unadjusted β (95% CI) 26.5 (− 0.9, 53.8); Adjusted β (95% CI) 20.9 (1.1, 40.7) respectively]. This implies that 1 unit (here % of fatty acids) change in mono trans fatty acid is associated with an average rise of triglycerides by 20 mg/dL.

**Table 3:** Regression analysis of trans fatty acids of C18 series

Fatty acids	TC	TG	HDL	TC/HDL	LDL	Apo A1	Apo B	hs CRP
18:1 trans								
β Unadjusted (95% CI)	− 8.9 (− 24.3, 6.5)	19.3 (− 12.0, 50.5)	− 2.2 (− 6.9, 2.4)	0 (− 0.5, 0.5)	− 13.8 (− 29.1, 1.4)	3.3 (− 9.4, 16.0)	− 7.9 (− 18.6, 2.7)	− 1 (− 5.4, 3.3)
β Adjusted (95% CI)^#^	− 14.1 (− 38.6, 10.4)	26.2 (− 3.4, 75.8)	− 4.3 (− 11.5, 2.9)	0.1 (− 0.7, 0.8)	− 21.9 (− 45.8, 2.1)	4.6 (− 15.3, 24.6)	− 11.2 (− 28.0, 5.7)	− 2.3 (− 9.5, 4.8)
18:2 trans								
β Unadjusted (95% CI)	− 5.4 (− 15.9, 5.1)	− 5.9 (− 27.2, 15.5)	− 2.9 (− 6.1, 0.3)	0.2 (− 0.1, 0.6)	− 2.8 (− 13.7, 8.1)	− 6.3 (− 15.3, 2.6)	− 3.2 (− 10.8, 4.4)	− 1.4 (− 4.5, 1.7)
β Adjusted (95% CI)^#^	− 2.1 (− 6.6, 2.5)	2.0 (− 7.0, 11.0)	− 1.0 (− 2.2, 0.2)	0.1 (− 0.0, 0.2)	− 1.3 (− 5.9, 3.3)	− 1.4 (− 5.0, 2.2)	0.5 (− 2.6, 3.6)	− 0.1 (− 0.5, 0.2)
18:3 trans								
β Unadjusted (95% CI)	− 0.1 (− 5.7, 5.4)	0.8 (− 10.5, 12.1)	1.0 (− 0.7, 2.7)	− 0.1 (− 0.3, 0.0)	− 0.3 (− 5.7, 5.2)	− 2.2 (− 6.7, 2.3)	− 0.9 (− 4.7, 2.9)	0.5 (− 1.0, 2.1)
β Adjusted (95% CI)^#^	− 1.5 (− 8.9, 6.0)	2.3 (− 12.7, 17.2)	0.8 (− 1.4, 2.9)	− 0.1 (− 0.4, 0.1)	− 3.2 (− 10.0, 3.6)	− 2.6 (− 8.2, 3.0)	− 1.8 (− 6.6, 2.9)	0.3 (− 1.7, 2.4)

TC, total cholesterol; TG, triglycerides; HDL, high-density lipoprotein cholesterol; LDL, low-density lipoprotein cholesterol; Apo A1, apolipoprotein A1; Apo B, apolipoprotein B; hs CRP, high sensitive c-reactive protein

* Values are significant (p < 0.05)

^#^Model adjusted with age, sex, bmi, diet, smoking and alcohol use

**Table 4:** Regression analysis of trans fatty acids in groups

Fatty acids	TC	TG	HDL	TC/HDL	LDL	Apo A1	Apo B	hs CRP
Mono trans								
β Unadjusted (95% CI)	− 1.0 (− 11.1, 9.2)	22.9 (2.6, 43.2)*	− 2.2 (− 5.2, 0.9)	0.1 (− 0.2, 0.5)	− 1.2 (− 11.9, 9.4)	0.7 (− 8.1, 9.6)	1.3 (− 6.1, 8.8)	− 1.1 (− 4.1, 2.0)
β Adjusted (95% CI)^#^	− 0.2 (− 9.2, 8.8)	20.4 (3.5, 37.3)*	− 0.8 (− 3.3, 1.7)	0 (− 0.2, 0.3)	− 4.0 (− 12.6, 4.7)	1.2 (− 5.8, 8.3)	− 0.6 (− 6.7, 5.6)	− 0.8 (− 3.4, 1.8)
Poly trans								
β Unadjusted (95% CI)	− 1.2 (− 5.9, 3.5)	− 0.6 (− 10.2, 9.0)	0.1 (− 1.3, 1.6)	− 0.1 (− 0.2, 0.1)	− 0.7 (− 5.4, 4.0)	− 2.8 (− 6.6, 1.0)	− 1.2 (− 4.5, 2.0)	0.1 (− 1.2, 1.5)
β Adjusted (95% CI)^#^	− 2.9 (− 10.7, 4.9)	2.4 (− 13.3, 18.2)	0 (− 2.2, 2.3)	− 0.1 (− 0.3, 0.2)	− 3.8 (− 11.0, 3.5)	− 4.1 (− 10.1, 1.8)	− 2.3 (− 7.4, 2.7)	0.1 (− 2.0, 2.3)
Total trans								
β Unadjusted (95% CI)	− 1.1 (− 5.2, 3.1)	3.4 (− 5.0, 11.8)	− 0.3 (− 1.5, 1.0)	0 (− 0.2, 0.1)	− 0.8 (− 5.0, 3.5)	− 2.2 (− 5.7, 1.3)	− 0.8 (− 3.8, 2.2)	− 0.1 (− 1.3, 1.1)
β Adjusted (95% CI)^#^	− 2.9 (− 12.8, 7.1)	9.5 (− 10.7, 29.6)	− 0.8 (− 3.7, 2.1)	− 0.1 (− 0.4, 0.3)	− 3.6 (− 13.2, 6.0)	− 5.6 (− 13.5, 2.2)	− 1.5 (− 8.2, 5.2)	0.1 (− 2.7, 3.0)

TC, total cholesterol; TG, triglycerides; HDL: high-density lipoprotein cholesterol; LDL, low-density lipoprotein cholesterol; Apo A1, apolipoprotein A1; Apo B, apolipoprotein B; hs CRP, high sensitive c-reactive protein

* Values are significant (p < 0.05)

^#^Model adjusted with age, sex, bmi, diet, smoking and alcohol use

**Table 5:** Regression analysis of individual trans fatty acids

Fatty acids	TC	TG	HDL	TC/HDL	LDL	Apo A1	Apo B	hs CRP
C16:1 trans								
β Unadjusted (95% CI)	5.1 (− 8.5, 18.7)	26.5 (− 0.9, 53.8)	− 2.2 (− 6.3, 1.9)	0.3 (− 0.2, 0.7)	10.6 (− 4.3, 25.4)	− 1.6 (− 14.0, 10.7)	10.1 (− 0.2, 20.4)	− 1.1 (− 5.3, 3.1)
β Adjusted (95% CI)^#^	3.4 (− 7.1, 13.9)	20.9 (1.1, 40.7)*	− 0.4 (− 3.3, 2.5)	0.1 (− 0.2, 0.4)	0.7 (− 9.4, 10.8)	0.2 (− 8.0, 8.4)	2.6 (− 4.5, 9.7)	− 0.3 (− 3.4, 2.7)
C18:1 11 *trans* (Vaccenic acid)								
β Unadjusted (95% CI)	− 22.1 (− 55.4, 11.2)	33.3 (− 34.5, 101.1)	− 0.4 (− 10.6, 9.7)	− 0.5 (− 1.6, 0.6)	− 21.1 (− 53.0, 10.8)	− 13 (− 39.4, 13.5)	− 7.6 (− 30.0, 14.7)	− 1.6 (− 10.7, 7.5)
β Adjusted (95% CI)^#^	− 25.6 (− 72.3, 21.1)	60.3 (− 34.1, 154.7)	0.3 (− 13.4, 14.1)	− 0.8 (− 2.3, 0.7)	− 25.6 (− 69.0, 17.8)	− 16.5 (− 52.3, 19.3)	− 4.8 (− 35.3, 25.7)	− 4.9 (− 17.8, 8.0)
C18:1 9 *trans* (Elaidic acid)								
β Unadjusted (95% CI)	− 5.3 (− 22.8, 12.2)	15.5 (− 20.1, 51.1)	− 2.8 (− 8.1, 2.5)	0.2 (− 0.4, 0.7)	− 12 (− 29.7, 5.7)	8.4 (− 6.3, 23.1)	− 8.3 (− 20.7, 4.0)	− 0.9 (− 6.0, 4.2)
β Adjusted (95% CI)^#^	− 11.4 (− 38.6, 15.8)	10.1 (− 45.1, 65.2)	− 5.2 (− 13.2, 2.8)	0.2 (− 0.6, 1.1)	− 20.4 (− 48.0, 7.1)	12.9 (− 9.8, 35.7)	− 14.4 (− 33.7, 4.9)	− 1.3 (− 9.5, 6.9)

TC, total cholesterol; TG, triglycerides; HDL, high-density lipoprotein cholesterol; LDL, low-density lipoprotein cholesterol; Apo A1, apolipoprotein A1; Apo B, apolipoprotein B; hs CRP, high sensitive c-reactive protein

* Values are significant (p < 0.05)

^#^Model adjusted with age, sex, bmi, diet, smoking and alcohol use

### Discussion

The diet among Indian population varies considerably with geographical location. Unlike western countries where majority of the population is dependent on packaged food, Indians are more experimental with their indigenous agricultural products. This gives rise to an enormous possibility of food categories and types, and their fatty acid content assessment is a labor intensive task, however, serum fatty acid profile reflects the recent intake through diet. Fasting serum/plasma is a preferred sample of choice for epidemiological studies to assess dietary fatty acid intake, we have assessed the association between fatty acids & cardiovascular risk markers in the present study.

Evaluation of the effect of C18:2 *trans* and C18:3 *trans* on cardiovascular risk markers through dietary studies was not found in literature. Majority of the studies have been conducted in western countries and have evaluated effect of C18:1 *trans*, as the major oil in their diet is monounsaturated fatty acid.

In our study, mono trans fatty acid appears to be independently associated with raised triglycerides. The major contributor of mono trans are the trans-palmitoleic (palmitelaidic), elaidic and vaccenic acid, of which trans palmitoleic was the major contributor for significant raise in serum triglycerides. The major source of trans-palmitoleic acid in the study population was milk which was reported to be consumed by almost all the participants. The trans palmitoleic acid has been found to be triglycerides lowering by [14]. Association with raised triglycerides in our study could be due to hepatic release of endogenous triglycerides as a result of excess intake of carbohydrate as it is customary in India to consume milk & its product in the form of sweet. It is worthwhile to be noted that the 95% confidence interval in the regression analysis was wide due to small sample size.

In conclusion poly trans and total trans were not found to be associated with any of the cardiovascular risk markers in our study. Mono trans showed significant association with triglycerides.

We did not find any association between trans fatty acid and risk markers probably due to inadequate sample size, however, in spite of all the limitations and shortcomings, our study is the first in India to evaluate the effect of serum trans fatty acids on risk markers of cardiovascular disease.

## Limitations

The study being cross-sectional in nature, could not derive a causative relationship between fatty acids and cardiovascular risk markers. The other limitation of our study is that dietary data was not collected which would have served as a validation for the fatty acids estimated in serum. Also the effect of fatty acids on various risk markers could have been evaluated after correcting with total energy intake.

## Additional file

**Additional file 1: Table S1.** Percent content of fatty acids in serum.

## Data Availability

The datasets used during the current study are available from the corresponding author on reasonable request.

## Abbreviations

CVD: cardiovascular disease
hs CRP: high-sensitive c-reactive protein
SFA: saturated fatty acid
MUFA: monounsaturated fatty acid
PUFA: polyunsaturated fatty acid
*t*: trans
