# Supplementary material for: Association of trans fatty acids with lipids and other cardiovascular risk factors in an Indian industrial population
Source: BMC Res Notes. 2019 Jun 17;12:342. doi: 10.1186/s13104-019-4352-7 (PMC6580624; doi:10.1186/s13104-019-4352-7)
Supplement: Supplementary file 1 — Additional file 1: Table S1. Percent content of fatty acids in serum. [file 13104_2019_4352_MOESM1_ESM.docx]

**Additional file 1: Table S1**: Percent content of fatty acids in serum

| **Fatty acids** | **Mean (SD)** | **Median (IQR)** |
| --- | --- | --- |
| C 12:0 (Lauric acid) | 0.75 (0.74) | 0.62 (0.18 - 1.00) |
| C 14:0 (Myristic acid) | 1.32 (0.44) | 1.26 (0.98 - 1.60) |
| C 15:0 (Pentadecanoic acid) | 0.12 (0.13) | 0.06 (0.02 - 0.21) |
| C 16:0 (Palmitc acid) | 24.49 (3.16) | 24.48 (22.72 - 26.04) |
| C 18:0 (Stearic acid) | 6.51 (1.18) | 6.67 (6.03 - 7.19) |
| Total SFA | 33.18 (3.63) | 33.17 (30.73 - 33.17) |
| C 14:1 *5cis* (n-9) | 0.03 (0.05) | 0.02 (0 - 0.041) |
| C 16:1 *7cis* (Palmitoleic acid) | 1.92 (1.07) | 1.79 (1.20 - 2.46) |
| C 18:1 *cis* (Oleic acid) | 20.04 (3.6) | 20.08 (17.07 - 22.69) |
| Total MUFA | 23.07 (4.11) | 22.82 (20.11 - 25.71) |
| C 18:2 *cis* (Linoleic acid) | 31.80 (5.05) | 31.68 (28.92 - 35.22) |
| C 18:3 n-6 (γ-Linolenic acid) | 0.08 (0.20) | 0.02 (0 - 0.07) |
| C 18:3 n-3 (α-Linolenic acid) | 0.15 (0.33) | 0.02 (0 - 0.13) |
| C 20:2 n-6 (Eicosadienoic acid) | 0.16 (0.15) | 0.12 (0 - 0.28) |
| C 20:3 n-6 (Dihomo-γ-linolenic acid) | 1.37 (1.72) | 1.35 (0.30 - 1.98) |
| C 20:3 (n-3) | 0.45 (0.62) | 0.23 (0.03 - 0.62) |
| C 20:4 n-6 (Arachidonic acid) | 5.43 (1.72) | 5.37 (4.51 - 6.53) |
| C 20:5 n-3 (Eicosapentaenoic acid) | 0.44 (0.46) | 0.32 (0.04 - 0.77) |
| C 22:6 n-3 (Docosahexaenoic acid) | 0.33 (0.49) | 0.04 (0 - 0.67) |
| Total PUFA | 42.02 (5.55) | 42.56 (38.73 - 45.77) |
| C 18:1 *trans* | 0.13 (0.29) | 0.02 (0-0.16) |
| C 18:2 *trans* | 0.20 (0.43) | 0.06 (0-0.21) |
| C 18:3 *trans* | 1.63 (0.83) | 1.52 (1.05 - 2.14) |
| Mono *trans* fatty acid | 0.67 (0.45) | 0.61 (0.49 - 0.72) |
| Poly *trans* fatty acid | 1.83 (0.97) | 1.67 (1.18 - 2.35) |
| Total *trans* fatty acid | 2.5 (1.10) | 2.36 (1.74 - 3.05) |
| C 16:1 *trans* (Palmitelaidic acid) | 0.53 (0.34) | 0.51 (0-0.60) |
| C 18:1 *9 trans* (Elaidic acid) | 0.08 (0.26) | 0.0 (0-0) |
| C 18:1 *11 trans* (Vaccenic acid) | 0.05 (0.14) | 0.0 (0-0.05) |

*SFA: Saturated fatty acid; MUFA: monounsaturated fatty acid, PUFA: polyunsaturated fatty acid,18:1 trans: C 18:1 9t + C 18:1 11t, mono trans fatty acids: C16:1 t + C 18:1 9t + C 18:1 11t; poly trans fatty acids: C 18:2 t + C 18:3 t; total trans fatty acid: mono trans + poly trans*
